# Supplementary material for: Development of monoclonal antibodies targeting the conserved fragment of hexon protein to detect different serotypes of human adenovirus
Source: Microbiol Spectr. 2024 Feb 22;12(4):e01816-23. doi: 10.1128/spectrum.01816-23 (PMC10986570; doi:10.1128/spectrum.01816-23)
Supplement: Supplemental material — Fig. S1 to S4. [file spectrum.01816-23-s0001.docx]

Supplementary Materials for

**Development of monoclonal antibodies targeting the conserved fragment of Hexon protein for broadly detecting different serotypes of human adenovirus**

This PDF file includes:

Supplementary Fig. 1 to Fig.4

**Supplementary Fig. 1**


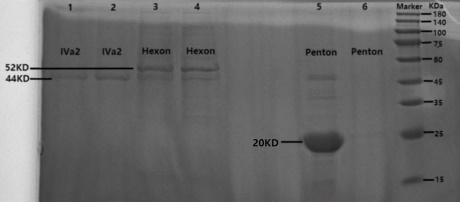


**Supplementary Fig. 1 SDS-PAGE analysis of purified recombinant protein**

(Lane 1) The precipitation of purified IVa2 protein. (Lane 2) The supernatant of purified IVa2 protein. (Lane 3) The precipitation of purified Hexon protein. (Lane 4) The supernatant of purified Hexon protein. (Lane 5) The precipitation of purified Penton protein. (Lane 6) The supernatant of purified Penton protein.

**Supplementary Fig. 2**


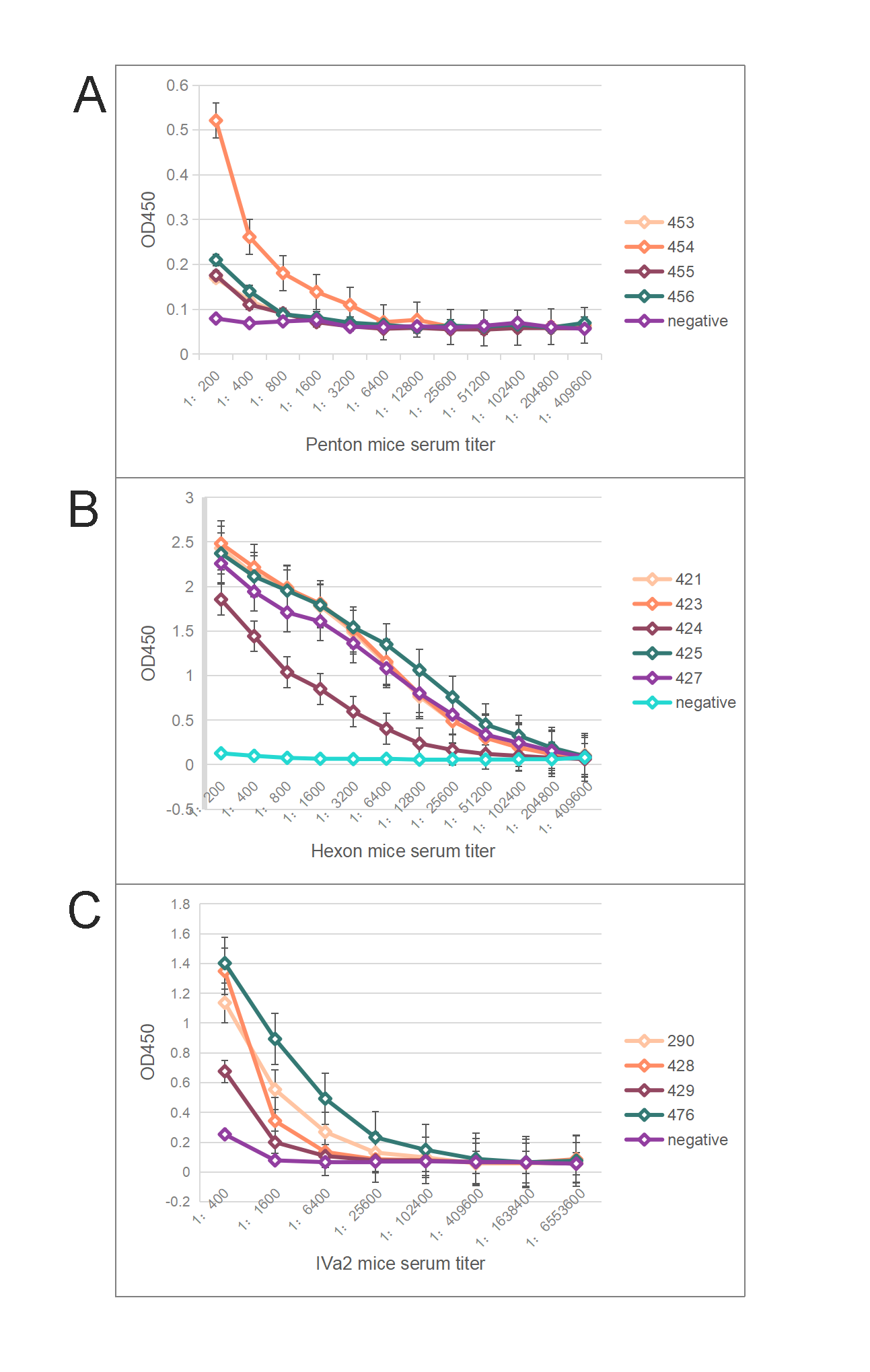


**Supplementary Fig.2. Serum titer identification from immunized mice**

1. Serum titers of mice immunized with Penton protein. (B) Serum titers of mice immunized with Hexon protein. (C) Serum titers of mice immunized with IVa2 protein.

**Supplementary Fig. 3**


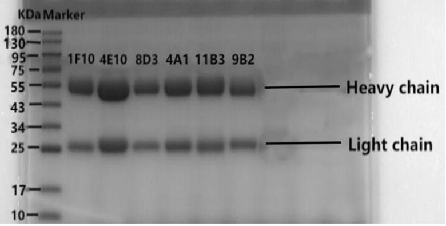


**Supplementary Fig. 3 Monoclonal antibodies purification**

SDS-PAGE analysis of purified 1F10, 4E10, 8D3, 4A1 and 11B3 antibodies specific to Hexon of HAdV and purified 9B2 antibodies specific to IVa2 of HAdV.

**Supplementary Fig.4**

**
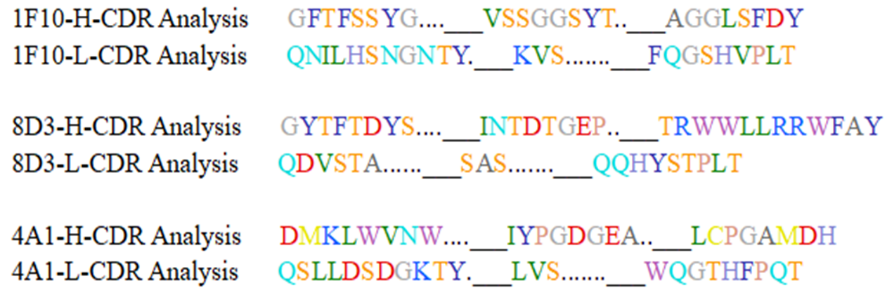
**

**Supplementary Fig.4 CDR sequences of 1F10, 8D3 and 4A1 monoclonal antibodies.**
